# Advancing the Evaluation of Risk-Stratified Colorectal Cancer Screening by Simulating Quantitative Fecal Hemoglobin Concentrations

**DOI:** 10.1177/23814683261440920

**Published:** 2026-05-12

**Authors:** Danica M. N. van den Berg, Luuk A. van Duuren, Lucie de Jonge, Iris Lansdorp-Vogelaar

**Affiliations:** Department of Public Health, Erasmus MC, University Medical Center, Rotterdam, The Netherlands; Department of Public Health, Erasmus MC, University Medical Center, Rotterdam, The Netherlands; Department of Public Health, Erasmus MC, University Medical Center, Rotterdam, The Netherlands; Department of Public Health, Erasmus MC, University Medical Center, Rotterdam, The Netherlands

**Keywords:** colorectal cancer, prior FIT results, risk-based screening, microsimulation, fecal hemoglobin

## Abstract

**Background.** Growing evidence indicates that fecal hemoglobin (f-Hb) concentrations from prior negative fecal immunochemical tests (FITs) can predict the future detection of colorectal cancer (CRC) and advanced neoplasia. As a result, there are opportunities for risk-based screening tailored to prior FIT outcomes. However, current CRC screening decision models do not explicitly simulate quantitative f-Hb concentrations. They simulate only a binary positive or negative test result based on the sensitivity and specificity of the underlying lesions, which makes it impossible to evaluate risk-based screening by f-Hb with these models. **Methods.** We extended our well-established MISCAN-Colon microsimulation model with a module that simulates quantitative f-Hb concentrations based on age, sex, and the presence of colorectal lesions. This module was based on a zero-inflated negative binomial mixed-effect model and calibrated and validated using observational data from the Dutch national CRC screening program between 2014 and 2020. The new MISCAN-Colon was then applied to a case study, in which we compared the effectiveness of uniform biennial screening intervals versus risk-based intervals (3 y after a FIT result of 0 µg/g, 2 y after >0–15 µg/g, and 1 y after >15–46.9 µg/g). **Results.** The updated model closely reflected real-world results, including positivity rate (observed: 4.7%, model: 4.3%), CRC detection rates (observed: 0.3%, model: 0.4%), f-Hb distributions, and longitudinal patterns across 3 screening rounds. Compared with biennial screening, the risk-based strategy resulted in slightly more CRC cases (+5%) and deaths (+11%) but required substantially fewer FITs (−28%) and colonoscopies (−13%). **Conclusions.** Integrating the f-Hb module into MISCAN-Colon enables explicit modeling of f-Hb concentrations and facilitates the evaluation of risk-based screening by prior f-Hb concentrations.

**Highlights:**

- In colorectal cancer screening (CRC), risk-based screening using fecal hemoglobin (f-Hb) from prior screening rounds has been suggested to improve the balance between the benefits and harms of screening. However, the added value of this approach has never been quantified because none of the existing decision models for CRC screening simulate f-Hb concentrations.
- We used data from the Dutch national CRC screening program to extend the widely used MISCAN-Colon microsimulation model to explicitly simulate an individual’s f-Hb concentration based on sex, age, and the presence of colorectal lesions.
- This model accurately reproduced cross-sectional and longitudinal associations between f-Hb concentration and screen-detected colorectal lesions, and model estimates showed that risk-based screening using f-Hb concentrations could be more efficient than current uniform screening.

## Graphical abstract

**Graphical abstract:**
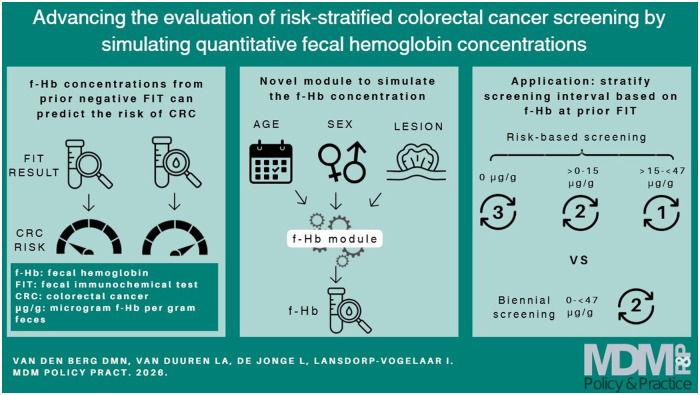

Effective screening is crucial for reducing both the incidence and mortality associated with colorectal cancer (CRC) through the early detection and prevention of CRC.^1,2^ In response, many countries across Europe have established a national CRC screening program, with the most using the fecal immunochemical test (FIT) for CRC detection.^3^ Although there are multiple FIT brands in use internationally, each with different analytical characteristics and measurement ranges,^4^ the underlying principle is the same: detecting fecal hemoglobin (f-Hb), which may indicate gastrointestinal bleeding from CRC or precancerous lesions. Individuals with an f-Hb concentration above a specified positivity threshold are typically referred for a diagnostic colonoscopy, whereas those with an f-Hb concentration below the cutoff are invited for FIT screening again after a fixed interval. In this way, FIT is used in a binary manner.

Prior studies, however, have demonstrated that individuals with f-Hb concentrations just below the FIT positivity threshold have a substantially higher risk of CRC compared with those with no detectable f-Hb in their stool.^5–10^ This suggests that FIT programs could be improved by implementing a screening policy with risk-tailored intervals or positivity cutoffs based on an individual’s prior FIT results. Two randomized controlled trials currently investigate such approaches.^11,12^ For example, the PERFECT-FIT trial assigns a 1-y interval for individuals with prior f-Hb >15 to 47 µg per gram of feces (µg/g), a 2-y interval for those with prior f-Hb >0 to 15 µg/g, and a 3-y interval otherwise.

However, it remains unclear exactly how CRC screening programs can be tailored to prior FIT results in order to maximize benefits like early cancer detection and reduced mortality while minimizing harms such as unnecessary colonoscopies, overdiagnosis, and patient burden, and it is infeasible to evaluate all potential strategies through clinical trials alone. Decision models are widely used to assess and optimize different screening approaches by simulating a virtual population undergoing screening.^13–15^ However, most decision models currently simulate FIT-based screening as a dichotomous (positive/negative) result based on the test’s sensitivity and specificity. To evaluate risk-based approaches that incorporate prior FIT results, these models must instead simulate quantitative f-Hb concentrations as the outcome of the FIT. To address this gap, prior work by Van Duuren et al.^16^ introduced a prototype module that is designed to handle data with many zero values (when no f-Hb is detected) while also accounting for the correlation between repeated FIT measurements within the same individual over time. Building on this foundation, our study aims to refine, calibrate, and validate this module and integrate it into the Microsimulation Screening Analysis for CRC (MISCAN-Colon) model.^17^ This will enable simulation of individual’s prior FIT result and allows for cost-effectiveness analyses of risk-based approaches using prior FIT results.

## Methods

In this study, we developed a module to simulate quantitative f-Hb concentrations in MISCAN-Colon based on age, sex, and presence of colorectal lesions using a zero-inflated negative binomial mixed-effect model. We calibrated and validated the model parameters using observational data from the Dutch national CRC screening program. Finally, as a case study, we used the module to explore the benefits and harms of risk-based intervals for FIT based on f-Hb concentration versus uniform biennial screening intervals.

### Data Source

The Dutch national CRC screening program targets individuals aged 55 to 75 y and offers biennial FIT screening to detect early signs of CRC. The FIT is designed to detect blood in stool samples by binding antibodies to f-Hb. Colonoscopy is performed for individuals with positive FIT results to confirm the presence of colorectal lesions or cancer. The program, which began in 2014, initially set the positivity threshold for colonoscopy referral at 15 µg/g but switched to a cutoff of 47 µg/g in the second half of 2014.^18^ The program’s data, such as FIT outcomes, colonoscopy results, and pathology diagnoses, are systematically recorded in ScreenIT, the national information system used for managing cancer screening data. The complete dataset included 7,950,767 FIT results from 3,909,700 individuals collected between 2014 and 2020 (Table 1). Between 2014 and 2020, individuals could undergo up to 4 screening rounds but most were screened only once or twice by 2020 because of the gradual expansion of the program. Overall, the positivity rate for FIT (proportion of positive FITs among all FITs) was 5%, while the detection rates (proportion of FITs with detected lesions among all FITs) for CRC, advanced adenomas, and nonadvanced adenomas were 0.3%, 2%, and 1%, respectively.

**Table 1:** Description of the Datasets Used for the Calibration and Validation of the f-Hb Module.^a^

	Calibration Dataset		Validation Dataset		Total	
Years	First half 2014		Mid-2014–2020		2014–2020	
Positivity cutoff	15 µg/g		47 µg/g		15 and 47 µg/g	
No. of individuals	126,255		3,783,445		3,909,700	
No. of analyzed FIT results	126,255		7,824,512		7,950,767	
Round 1	126,255	(100%)	3,723,855	(48%)	3,850,110	(48%)
Round 2	0		2,494,294	(32%)	2,494,294	(32%)
Round 3	0		1,411,340	(18%)	1,411,340	(18%)
Round 4	0		195,023	(2%)	195,023	(2%)
Positive FITs	14,440	(11%)	366,446	(5%)	383,419	(5%)
Detected colorectal cancer cases	941	(0.7%)	21,364	(0.3%)	22,305	(0.3%)
Detected advanced adenomas^b^	4,815	(4%)	119,004	(2%)	123,819	(2%)
Detected nonadvanced adenomas^b^	2,758	(2%)	78,127	(1%)	80,885	(1%)
f-Hb result of 0 µg/g	83,080	(66%)	6,725,120	(86%)	6,808,200	(86%)

f-Hb, fecal hemoglobin; FIT, fecal immunochemical test; µg/g, microgram f-Hb per gram feces.

a The data originate from the Dutch national colorectal cancer (CRC) screening program, which targets individuals aged 55 to 75 y for biennial screening. Rounds 1 to 4 correspond to the first through fourth screening episodes of an individual.

b Nonadvanced adenomas were defined as adenomas <10 mm in size, whereas adenomas ≥10 were classified as advanced adenomas.

The distribution of f-Hb concentrations in the screened population is highly skewed, with a large proportion of individuals having undetectable or very low f-Hb concentrations (Supplementary Figure 1). However, among those with detectable f-Hb, concentrations vary widely. Notably, the distribution exhibits 2 distinct peaks: the first occurs at 0 µg/g, while the second is observed at higher f-Hb concentrations, typically between 200 and 300 µg/g. This bimodal distribution can be attributed to 2 distinct groups: individuals with and without advanced neoplasia. The first peak, reflecting low f-Hb concentrations, is likely to primarily originate from individuals without advanced neoplasia who experience minimal or no bleeding. In contrast, the second peak is more likely to come from individuals who have advanced neoplasia and experience higher bleeding levels (Supplementary Figure 2). In addition, the second peak may be influenced by a phenomenon in which high f-Hb concentrations saturate the antibody-binding sites in the FIT, resulting in an underestimation of the actual f-Hb concentrations. This is known as the prozone effect. Consequently, some individuals with very high f-Hb concentrations may receive falsely low readings, leading to an accumulation of cases in this range.

The full dataset was split into 2 separate sets: one for model calibration and the other for model validation (Table 1 and Supplementary Figure 1). For calibration, we used data (*n* = 126,255) from the Dutch national screening program collected during the first half of 2014, with a positivity cutoff of 15 µg/g. For model validation, we used Dutch CRC screening data from mid-2014 to 2020, with a positivity cutoff of 47 µg/g. This validation dataset included 7,824,512 FIT results from 3,783,445 individuals, with up to 4 screening rounds per person. This division was made for 2 main reasons. First, to ensure that internal model validation was performed independently of the data used for model calibration and development, avoiding overfitting and enabling an unbiased evaluation of model performance. Second, the data of the first half of 2014 includes colonoscopy outcomes of individuals with a FIT result greater than 15 µg/g, whereas the remaining data include only these outcomes for results above the current 47 µg/g cutoff. Therefore, the model was calibrated across a larger range of values.

### MISCAN-Colon

MISCAN-Colon is a stochastic microsimulation model for CRC screening developed by the department of Public Health at the Erasmus Medical Center, implemented in Python 3. The model consists of 3 modules: demography, natural history, and screening.^17,19^ In the demography module, MISCAN-Colon utilizes birth and life tables to replicate a population by assigning each simulated individual a date of birth and a date of death from other causes than CRC. As simulated individuals age, they may develop 1 or more adenomas (benign lesions in the colon). This is simulated in MISCAN-Colon’s natural history module. Adenomas can be progressive or nonprogressive and may grow in size from small (≤5 mm) to medium (6–9 mm) to large (≥10 mm). Only progressive adenomas have the potential to develop into preclinical cancer, which progresses through stages I to IV. CRC can be diagnosed at any of these stages due to the appearance of symptoms. In the screening module, screening is added to the simulation. Consequently, some simulated life histories are modified since cancers will be prevented through the detection and removal of adenomas, while other cancers will be identified at an earlier stage, leading to better survival outcomes. In addition, the model can evaluate the harms of screening, including the number of false-positive test results, complications from colonoscopy, increased detection of precursor lesions that would not have progressed to CRC, and false-negative test results.

The parameters in the natural history model were calibrated separately for males and females to align with the age-, stage-, and localization-specific CRC incidence data from the Netherlands Cancer Registry (2009–2013),^20^ as well as the age-specific prevalence of adenomas and the distribution of the number of adenomas per individual based on findings from autopsy and colonoscopy studies.^21–30^ Although the latter studies are older and derived from other countries, adjustments were made to account for differences in CRC incidence across populations.

### f-Hb Module

**Figure 1:**
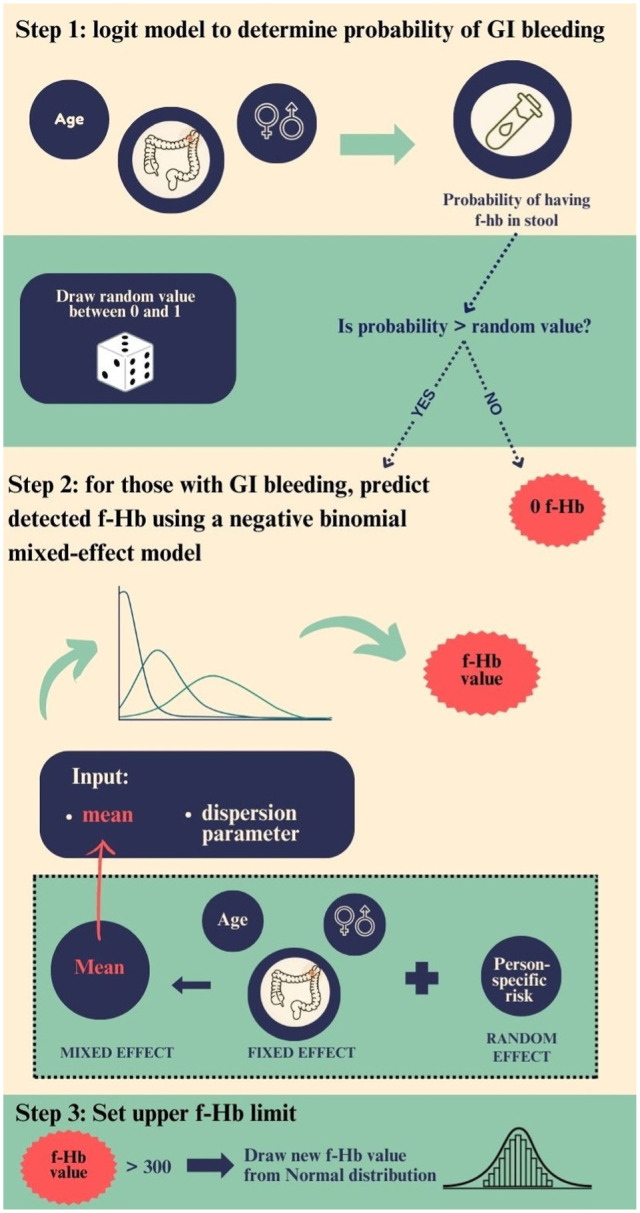
Infographic on the f-Hb module in MISCAN-Colon, which simulates the f-Hb concentrations based on age, sex, and underlying lesions. f-Hb, fecal hemoglobin concentration; GI, gastrointestinal.

We modeled quantitative f-Hb concentrations using a zero-inflated negative binomial mixed-effects model, depending on age, sex, and the presence and type of lesion (Figure 1).^31^ Three types of lesions were distinguished: nonadvanced adenoma (<10 mm in size), advanced adenoma (≥10 mm), or CRC. For individuals with multiple lesions, the module considers only the most advanced lesion. We chose a zero-inflated negative binomial mixed-effect model for 3 main reasons. First, the zero inflation allows us to account for the large number of screening tests in which no f-Hb is detected. Second, this model can handle the wide variation we see in f-Hb concentrations among people with nonzero results. Third, it takes into account that test results from the same person over time are correlated, rather than completely independent.

The module consisted of 3 parts:

- Zero-inflated component: A logistic regression estimates the probability that a test result is zero based on age, sex, and the most advanced lesion.
- Negative binomial component: For tests not classified as zeros, f-Hb concentrations are modeled using a negative binomial mixed-effects model. The mean of the negative binomial distribution depends on fixed effects for age, sex, and lesion status, which have the same effect across all individuals, and an individual-specific random intercept representing a person’s underlying risk of bleeding, which is assumed constant across all FIT tests for that individual.
- Random-effects component: This component induces the correlation between repeated f-Hb measurements from the same individual, which we assume is constant over time.

Because the test buffer has a maximum binding capacity, simulated f-Hb concentrations greater than 300 µg/g were adjusted. In such cases, the original concentration was replaced by a random draw from a normal distribution centered around the peak observed in the upper tail of the empirical f-Hb distribution (Supplementary Figures 1C, D and Figure 2). This approach ensures that extreme values are not unrealistically high and that the simulated f-Hb distribution reproduces the bimodal pattern seen in the real-world data.

Even when lesions are present, the module can still generate zero values, representing false-negative test results or concentrations below the detection threshold. Moreover, the module allows nonzero f-Hb in individuals without lesions, reflecting bleeding from nonneoplastic sources, such as hemorrhoids. The mathematical formulas for the f-Hb module are provided in Supplement A.

### Model Calibration

Sixteen unknown parameters of the f-Hb module were calibrated (Supplement A). From the calibration data, positivity and detection rates for CRC, advanced adenomas, and nonadvanced adenomas were derived for (hypothetical) positivity thresholds of 15, 20, 30, 40, 50, and 60 µg/g (Supplementary Table 1). The MISCAN-Colon model with the f-Hb module was calibrated against these rates by replicating the screened population. For model calibration, we assumed complete adherence to FIT screening, diagnostic colonoscopy, and surveillance. To adjust for nonadherence to diagnostic colonoscopy in the observed data, detection rates were adjusted accordingly. The calibration process for the parameters of the zero-inflated negative binomial mixed-effect model (14/16 parameters) was conducted using the Nelder–Mead Simplex algorithm, which is a numerical optimization method used to find the best-fitting parameter set by minimizing the difference between the simulated and observed data.^32^ The mean and the variance of the normal distribution (2/16 parameters), which was used to account for a maximum binding capacity of the test buffer, were manually calibrated so that the simulated f-Hb distribution matched the observed bimodal pattern (Supplementary Figure 1C, D).

### Model Validation

To validate the f-Hb module, MISCAN-Colon replicated the Dutch CRC screening program from mid-2014 to 2020 using actual participation rates. Model performance was evaluated through cross-sectional and longitudinal comparisons with observed screening data.

#### Cross-sectional validation

- The simulated distribution of f-Hb concentrations from mid-2014 to 2020 was compared with the observed distribution.
- The model-estimated positivity and detection rates at a 47 µg/g f-Hb cutoff were compared with observed rates from mid-2014 to 2020, stratified by screening round and colonoscopy finding (nonadvanced adenomas, advanced adenomas, CRC).
- The model-estimated positivity rate and detection rates for nonadvanced adenomas, advanced adenomas, and CRC from MISCAN-Colon simulations with and without the f-Hb module were compared to ensure that the new model was consistent with the old for uniform screening scenarios.

#### Longitudinal validation

- Mean f-Hb concentrations in screening round 3, stratified by f-Hb in rounds 1 and 2, were compared with observed data.
- Positivity rates in screening round 3, stratified by f-Hb in rounds 1 and 2, were compared with observed data.
- Detection rates for CRC and advanced neoplasia (CRC + advanced adenomas) in screening round 3, stratified by f-Hb concentrations in rounds 1 and 2, were compared with observed data.
- The intraclass correlation coefficient (ICC) is a measure to quantify the proportion of the total variance in f-Hb concentrations that is explained by differences between individuals, instead of differences caused by chance. It is calculated as the between-individual variance (the variance of the average f-Hb concentrations across individuals) divided by the total variance (sum of between-individual variance and within-individual variance). An ICC close to 1 means that most variation comes from differences between individuals, whereas an ICC close to 0 suggests that most of the variation is random. We compared the ICC of the modeled population to the ICC of observed data.

### Application: Model Outcomes Risk-Based Screening versus Uniform Screening

To illustrate the application of MISCAN-Colon with the f-Hb module, we simulated a cohort of 10 million individuals born in the Netherlands in 1968 and used the new model to evaluate 2 FIT screening strategies: 1) a uniform, biennial FIT screening program and 2) a risk-based screening program mirroring the design of the PERFECT-FIT trial.^11^ In the uniform strategy, all individuals were invited for FIT screening every 2 y from ages 55 to 75 y. In risk-based FIT screening, individuals aged 55 to 75 y were invited for FIT screening with intervals based on their prior negative FIT results. Those with 0 µg/g received a new screening invitation after 3 y, individuals with >0 to 15 µg/g after 2 y, and those with >15 to 46.9 µg/g after 1 y. In both uniform and risk-based screening, an f-Hb concentration of ≥47 µg/g was considered a positive FIT result, leading to a referral for diagnostic colonoscopy. The follow-up after adenoma detection for both screening strategies was modeled based on the current Dutch surveillance guidelines.^33^ We assumed 100% adherence to FIT screening, diagnostic colonoscopy, and surveillance colonoscopy. For each strategy, the model calculated lifetime projections for CRC incidence, CRC-related deaths, and the number of FITs and colonoscopies needed.

## Results

### Model Calibration

The parameters of the f-Hb module suggest that older individuals, males, and those with more advanced lesions have a higher likelihood of bleeding (Table 2). Among individuals with bleeding, higher quantitative f-Hb concentrations were observed in older individuals, males, and in those with an adenoma or CRC. The model-estimated positivity and detection rates fall within or are near the confidence intervals of the calibration targets (Supplementary Figure 3).

**Table 2:** Calibrated Parameters of the f-Hb Module Used for the Prediction of Hemoglobin Concentrations in MISCAN-Colon.

Model parameter	Calibrated value
Zero inflation (α)	
Intercept	2.400
Age	−0.017
Male	−0.277
Nonadvanced adenoma	−1.300
Advanced adenoma	−3.000
Colorectal cancer	−6.184
Fixed effect (β)	
Intercept	1.094
Age	0.014
Male	0.295
Nonadvanced adenoma	1.185
Advanced adenoma	2.789
Colorectal cancer	6.120
Random effect (b)	
Mean	0
Standard deviation (σ_b_)	0.114
Dispersion (θ)	0.202
Normal distribution f-Hb >300 µg/g	
Mean (µ_upper)_	225
Dispersion (σ_upper)_	25

f-Hb, fecal hemoglobin.

### Model Validation

The distribution of simulated f-Hb concentrations closely mirrored the observed distribution, with both showing 0 µg/g at the 50th, 75^th^, and 80th percentiles, indicating a large proportion of individuals with undetectable f-Hb concentrations (Supplementary Table 2). Beyond the 75th percentile, absolute differences did not exceed 5 µg/g, and the distribution of nonzero f-Hb concentrations revealed substantial overlap between the observed and simulated data, further demonstrating the strong alignment of f-Hb concentration distributions at higher levels (Supplementary Table 2 and Supplementary Figure 4).

The positivity and detection rates derived from the MISCAN-Colon model with the f-Hb module were consistent with those in the observational data as well as with the rates from the binary MISCAN-Colon model (Supplementary Figure 5). The average positivity rate across all screening rounds was 4.3% with the quantitative model, slightly lower than the 4.7% and 4.9% observed in the observational data and binary model, respectively. For detection rates, the quantitative model estimated averages of 0.4% for CRC, 2.0% for advanced adenomas, and 1.0% for nonadvanced adenomas. In comparison, the observational data reported rates of 0.3%, 1.5%, and 1.0%, while the binary model estimated rates of 0.5%, 1.8%, and 1.1%. Although the differences in positivity and detection rates between the quantitative model outputs and observational data were small (≤0.5 percentage points) on average across all screening rounds, larger deviations emerged for some specific screening rounds. The quantitative FIT model underestimated the positivity rate in round 1 by 0.9 percentage points compared with the observational data (4.5% v. 5.4%), but differences in subsequent rounds were less than 0.3 percentage points. For the detection rate, the largest deviation was observed for advanced adenomas in the second round (quantitative model: 1.9%, observed: 1.1%).

The f-Hb module reproduced key longitudinal patterns in mean f-Hb concentration, positivity rate, and detection rate across screening rounds. In both the observed and simulated data, the mean f-Hb concentration and positivity rate in round 3 increased with higher f-Hb concentrations in rounds 1 and/or 2 (Supplementary Figures 6–7). Overall, the module slightly underestimated mean f-Hb concentrations and positivity rates in round 3 compared with observed data. In addition, in both the observed and simulated data, detection rates for advanced neoplasia and CRC in the third screening round increased with higher f-Hb concentrations in rounds 1 and 2 (Supplementary Figures 8–9). For example, in the observed data, advanced neoplasia detection in round 3 increased from 1% among participants with 0 µg/g in both prior rounds to 22% among those with >30 µg/g in both rounds; in the model, the corresponding increase was from 1% to 19%. The ICC in the observed data was 0.06. This means that only 6% of the total variation in FIT results is attributable to systematic differences in bleeding between individuals, whereas 94% of the variance arises from differences between repeated FIT results within the same individual. Similarly, the ICC for the simulated f-Hb concentrations was very low, at 0.03.

### Application: Model Outcomes Risk-Based Screening versus Uniform Screening

Without screening, the MISCAN-Colon model estimated 86 CRC cases and 38 CRC deaths per 1,000 individuals over their lifetime (Table 3). Uniform biennial screening from ages 55 to 75 y would prevent 29 of those cases and 20 of the deaths compared with a scenario without screening. To achieve this, the model estimated that 8,745 FITs and 511 colonoscopies would be required. The risk-based strategy would require 28% fewer FITs and 13% fewer colonoscopies compared with uniform screening. However, this strategy would also be slightly less effective, preventing 5% fewer cases and 11% fewer deaths per 1,000 individuals.

**Table 3:** Comparative outcomes for No Screening, Uniform Biennial Screening from ages 55 to 75 y, and Risk-Based Screening Based on Fecal Hemoglobin (f-Hb) Concentration from Ages 55 to 75 y per 1,000 Simulated Individuals Born in 1968.

	No Screening	Uniform Biennial Screening	Risk-Based Screening^a^
CRC cases	86	57 (ref)	60 (+5%)
CRC deaths	38	18 (ref)	20 (+11%)
No. of FITs	0	8,745 (ref)	6,327 (−28%)
No. of colonoscopies	0	511 (ref)	443 (−13%)

CRC, colorectal cancer, FIT, fecal immunochemical test; µg/g f-Hb: microgram fecal hemoglobin per gram feces.

a In the risk-based model, individuals are invited for screening at 3-y intervals for 0 µg/g f-Hb, 2-y intervals for >0 to 15 µg/g f-Hb, and annually for >15 to 46.9 µg/g f-Hb.

## Discussion

Our study aimed to enhance the CRC screening evaluation by incorporating quantitative f-Hb concentrations into the MISCAN-Colon model, which previously simulated only binary FIT outcomes. We developed a module using a zero-inflated negative binomial mixed-effect model that accurately replicates f-Hb concentrations observed in the Dutch population. Using the new MISCAN-Colon model with quantitative FIT, we explored the effects of uniform biennial screening versus a risk-based approach that tailors screening intervals based on individual f-Hb concentrations mirroring the PERFECT-FIT trial protocol. Our results suggested that while this protocol prevented fewer CRC cases (+5%) and deaths (+11%), it substantially reduced the number of FITs (−28%) and colonoscopies (−13%) required.

The screening component of this new variant of MISCAN-Colon features a novel structure compared to existing microsimulation models for CRC. This enables a more nuanced integration of risk factors such as age and sex into the simulation of f-Hb concentrations and the determination of FIT positivity. Small discrepancies between the simulated and observed positivity and detection rates emerged during validation, potentially arising from assumptions about the underlying distribution of f-Hb concentrations. The model’s distribution may not fully capture the complexity of real-world data, which can be influenced by unaccounted factors such as comorbidities, lifestyle factors (e.g., smoking and diet), or measurement variability.

We modeled quantitative f-Hb concentrations using a zero-inflated negative binomial mixed-effects model. This approach effectively captures the high number of zero test results, the variability in nonzero concentrations, and the correlation of measurements within individuals over time. Although the negative binomial distribution is inherently discrete, it offers a flexible and interpretable way to approximate the continuous distribution of f-Hb observed in real data. While comparable continuous alternatives exist, such as zero-inflated log-normal or gamma mixed-effects models, these models are less flexible in modeling overdispersion and harder to interpret. The flexible structure of the f-Hb module also facilitates the integration of additional risk factors into the existing framework. As more evidence becomes available regarding the relationship between these factors and f-Hb concentrations, they can be incorporated into the model, either through the zero-inflation component or as fixed effects.

The results of the calibrated f-Hb module suggest that higher age and male sex increase both the likelihood of a nonzero f-Hb concentration and the quantitative value of f-Hb in the Dutch population. These findings align with international findings, which show that men and older individuals generally have higher f-Hb concentrations.^34,35^ Furthermore, the module simulates higher and more frequent nonzero quantitative f-Hb concentrations for individuals with more progressive lesions, such as advanced adenomas or CRC. This is consistent with the results reported by Navarro et al.,^36^ which observed a progressive increase in the proportion of patients with CRC or high-risk adenomas across quartiles of f-Hb concentration. In addition, a recent meta-analysis reported that elevated f-Hb concentrations are strongly associated with the detection of colorectal neoplasia in the subsequent screening round.^37^ Our findings suggest that the f-Hb module also captures this positive correlation between f-Hb concentrations in rounds 1 and 2 and those in round 3.

The strength of this study lies in the use of a large individual-level dataset for model development and validation with 2 different cutoffs (15 and 47 µg/g). The availability of data with a 15-µg/g cutoff allowed us to calibrate the model's performance in the range above 15 µg/g. This is particularly relevant for future evaluations of risk-based screening strategies that may involve the stratification of individuals with FIT concentrations below the standard 47 µg/g cutoff. Importantly, because the module was developed using the dataset with a 15 µg/g cutoff, it was independently validated using the dataset based on the 47 µg/g threshold, ensuring a clear separation between the calibration and validation datasets.

A limitation of our study is that the f-Hb module was calibrated using cross-sectional, first-round screening data because no individuals had more than 1 FIT test result using the cutoff level of 15 µg/g. Consequently, the random effects parameters primarily accounted for unexplained variability between individuals, rather than capturing longitudinal f-Hb patterns. However, the very low ICC in the observed data suggests that the majority of the variation occurs within individuals over time rather than between them. This suggests that the random effects in the data are minimal. In addition, when validating the longitudinal patterns, our module successfully captured an increase in mean f-Hb concentration, positivity rate, and detection rate in screening round 3 with increasing f-Hb concentrations in screening round 1 and 2. Nevertheless, the correlation between both the mean f-Hb concentration and positivity rate in round 3 and the f-Hb concentration in prior rounds was lower in our model compared with the data. Since the detection rates in round 3 were in line with the data when stratified by prior rounds, it could suggest that the module underrepresents systematic non–lesion-related bleeding events, whereas patterns associated with adenomas or CRC are well captured. This would also explain the slightly lower ICC in the model compared with data (0.03 v. 0.06). Another contributing factor to the underestimation may be the overall increase in positivity rates observed in recent years across all age groups in the Dutch CRC screening program without a similar increase in detection rate.^38^

Second, the f-Hb module is calibrated within the MISCAN-Colon framework and is therefore conditional on the model’s natural history assumptions for lesion development and progression. However, estimating an f-Hb module entirely outside the microsimulation would be challenging. Participants in FIT screening underwent a colonoscopy only after a positive FIT. As such, it is unknown whether individuals with f-Hb concentrations below the positivity threshold have lesions. By calibrating the f-Hb module within MISCAN-Colon, we had information on the underlying lesions of all individuals. However, it also means that the estimated associations should be interpreted as conditional on the assumptions underlying MISCAN-Colon. Nevertheless, our module validated well against observed data, providing strong evidence of its performance. That said, further validation is needed due to the relatively small sample size of individuals with more than 3 FIT results.

Third, the f-Hb module currently simulates the f-Hb concentration based on the most advanced lesion. However, it is plausible that individuals with multiple adenomas may exhibit higher f-Hb concentrations compared with those with only a single adenoma. While the module does not yet account for the number of adenomas, incorporating this as an additional covariate could enhance the model’s accuracy and is an important direction for future development.

The parameters of the f-Hb module were calibrated to Dutch data, which may not be directly applicable to other countries. Prior research indicates that f-Hb concentrations vary with age and sex, being higher in men and older individuals. However, the extent of this variation differs across countries.^33,35^ In addition, the Netherlands uses the FOB-Gold, whereas most other countries use the OC-Sensor test. These 2 tests are known to yield different f-Hb distributions.^39^ Consequently, while the module’s structure is likely reusable, its parameters may require adjustment or recalibration to accurately represent f-Hb distributions in other regions and for different tests. Nevertheless, the module explicitly links the f-Hb concentration to the presence and type of colorectal lesions. Therefore, in countries with higher (or lower) CRC incidence, where advanced precursor lesions are also more (or less) prevalent, the modeled f-Hb distribution will naturally shift toward higher (lower) concentrations. This allows the module to adapt meaningfully to different epidemiological contexts.

The f-Hb module has significant implications for advancing personalized CRC screening. In our case study, we focused on risk-based screening intervals based on individual f-Hb concentrations, but the module offers much broader possibilities. Beyond adjusting invitation intervals, the module can also evaluate risk-based stopping ages or positivity cutoff values for FIT, using age, sex, and prior f-Hb results, and compare them to various uniform strategies. By tailoring screening strategies, the module can help target high-risk individuals while reducing unnecessary screenings for low-risk groups.

While promising in concept, the results of risk-based screening by f-Hb in our case study may seem disappointing, as they suggest a reduction in the number of CRC cases and deaths prevented compared with uniform screening. However, this conclusion should not be generalized. In this analysis, we chose a scenario in which the screening interval for the largest group, the low-risk individuals, was extended by 1 y. While an extended interval reduces the frequency of unnecessary screenings, it may also delay the detection of adenomas or cancers in these low-risk individuals, potentially allowing the disease to progress before being detected. Different approaches to risk-based screening based on prior f-Hb concentrations could have yielded different outcomes, and the strategy chosen here is merely intended as an illustration. For example, it is conceivable that shortening the interval or lowering the positivity threshold solely for intermediate- and high-risk groups could improve CRC mortality outcomes relative to uniform screening.

To conclude, the integration of the f-Hb module into the MISCAN-Colon model marks an important step forward toward the evaluation of risk-based CRC screening by prior screening history. This module opens the door to performing (cost-)effectiveness analyses of risk-based screening based on prior FIT results. This enables the optimization of such screening strategies in the future, paving the way for more precise, efficient, and individualized approaches to the early detection of CRC.

## Supplemental Material

sj-docx-1-mpp-10.1177_23814683261440920 – Supplemental material for Advancing the Evaluation of Risk-Stratified Colorectal Cancer Screening by Simulating Quantitative Fecal Hemoglobin Concentrations Supplemental material, sj-docx-1-mpp-10.1177_23814683261440920 for Advancing the Evaluation of Risk-Stratified Colorectal Cancer Screening by Simulating Quantitative Fecal Hemoglobin Concentrations by Danica M. N. van den Berg, Luuk A. van Duuren, Lucie de Jonge and Iris Lansdorp-Vogelaar in MDM Policy & Practice
