# Supplementary material for: Advancing the Evaluation of Risk-Stratified Colorectal Cancer Screening by Simulating Quantitative Fecal Hemoglobin Concentrations
Source: MDM Policy Pract. 2026 May 12;11(1):23814683261440920. doi: 10.1177/23814683261440920 (PMC13172669; doi:10.1177/23814683261440920)
Supplement: sj-docx-1-mpp-10.1177_23814683261440920 – Supplemental material for Advancing the Evaluation of Risk-Stratified Colorectal Cancer Screening by Simulating Quantitative Fecal Hemoglobin Concentrations [file sj-docx-1-mpp-10.1177_23814683261440920.docx]

**Supplementary material**

| 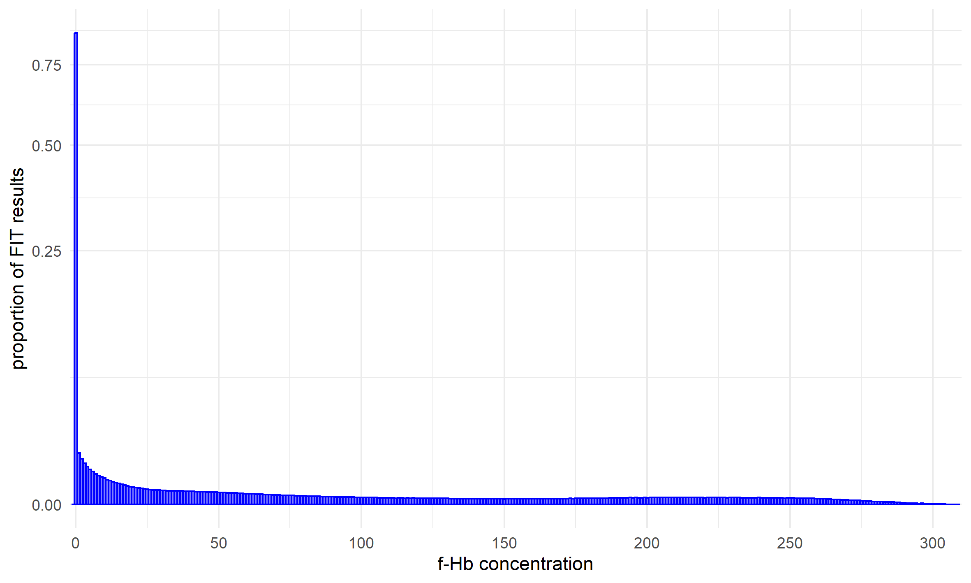  A) | 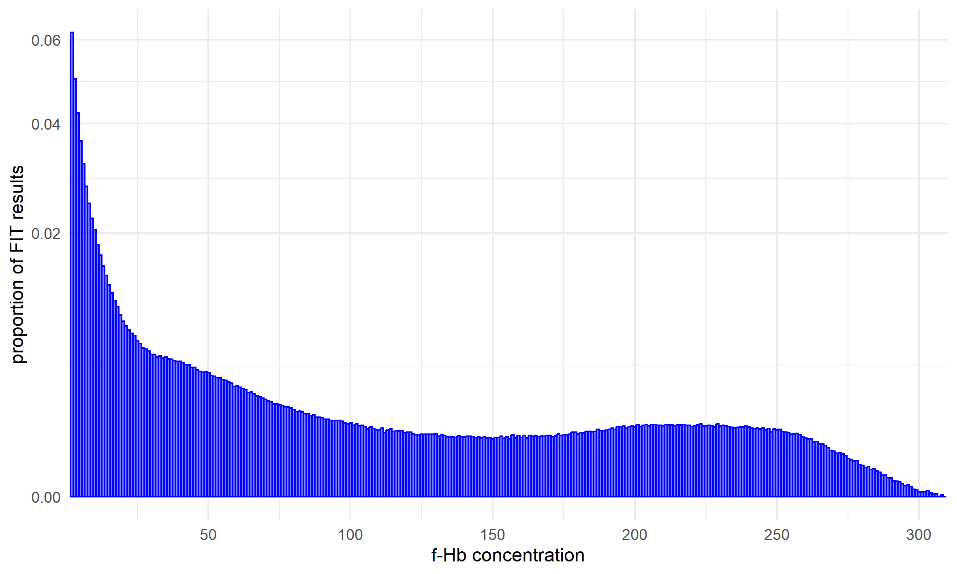  B) |
| --- | --- |
| 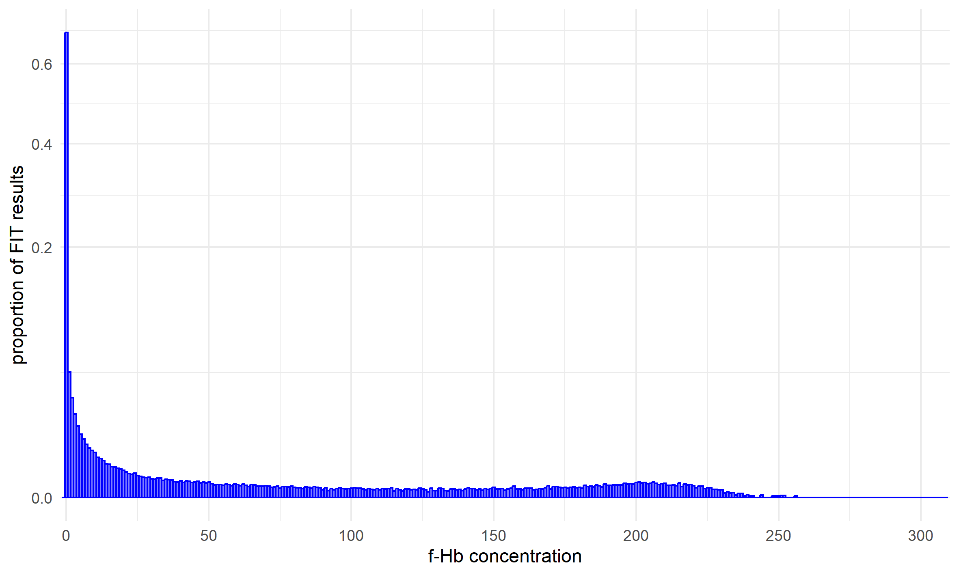  C) | 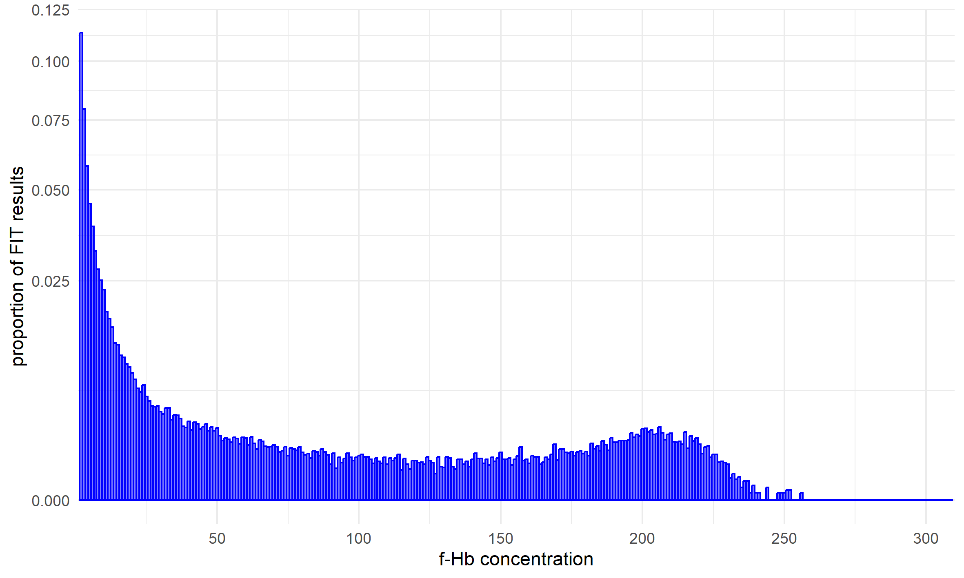  D) |
| 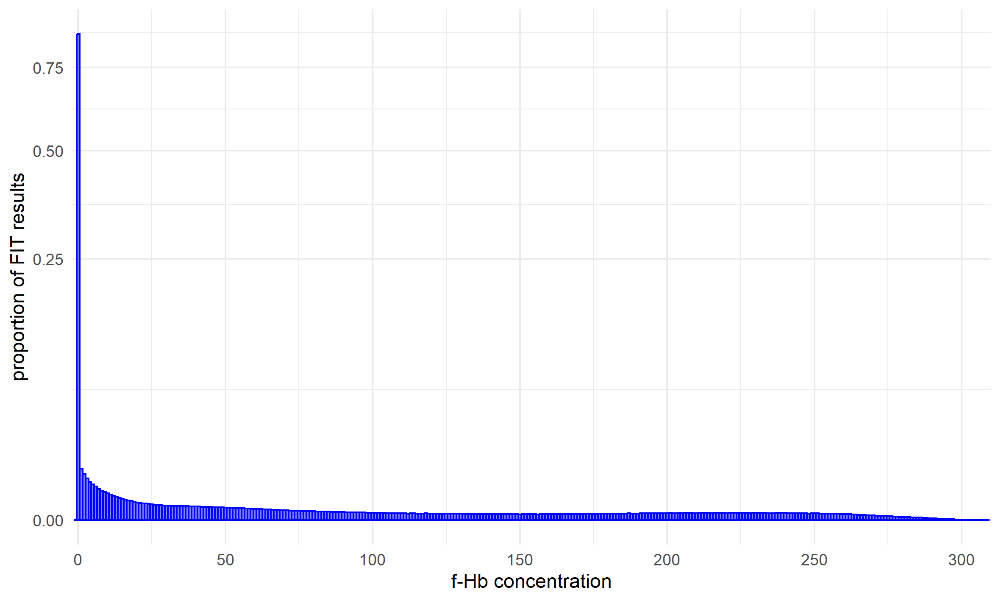  E) | 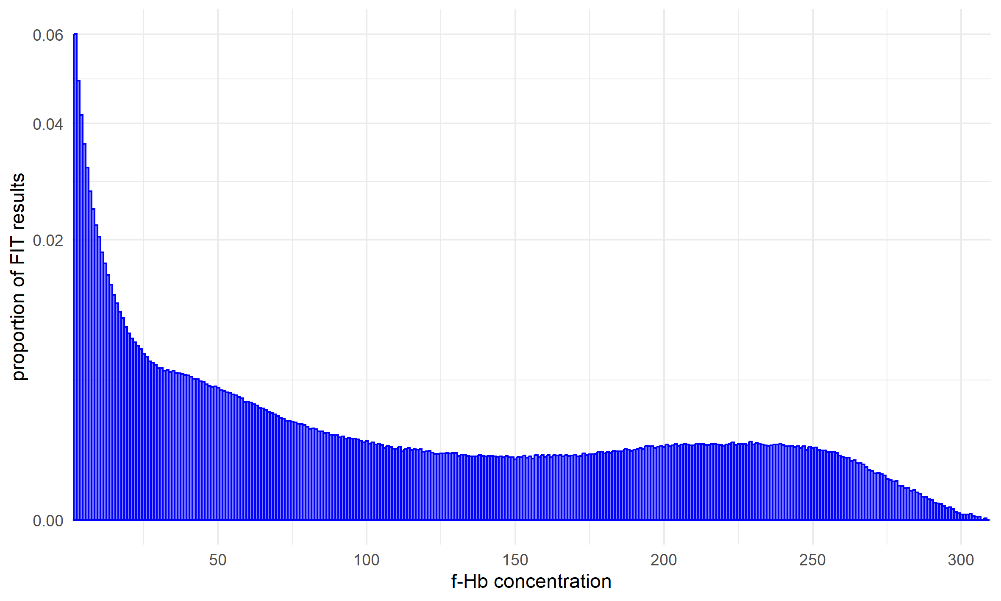  F) |

**Supplementary Figure 1** Distribution of fecal hemoglobin concentrations from the Dutch national CRC screening program, showing:
A) The full dataset from 2014 to 2020.
B) The full dataset including only f-Hb values strictly greater than 0,
C) The dataset that consists of tests in the first half of 2014, with a positivity cutoff of 15 µg/g. This dataset is used to fit the model parameters of the f-Hb module.
D) The dataset that consists of tests in the first half of 2014, with a positivity cutoff of 15 µg/g and only f-Hb values strictly greater than 0.
E) The dataset that consists of tests from mid-2014 to 2020, with a positivity cutoff of 47 µg/g. This dataset is used to check whether the model predictions match independent data after calibration.
F) The dataset that consists of tests from mid-2014 to 2020, with a positivity cutoff of 47 µg/g and only f-Hb values strictly greater than 0.
Abbreviations: FIT: fecal immunochemical test, f-Hb: fecal hemoglobin; µg/g. Microgram per gram; CRC, colorectal cancer


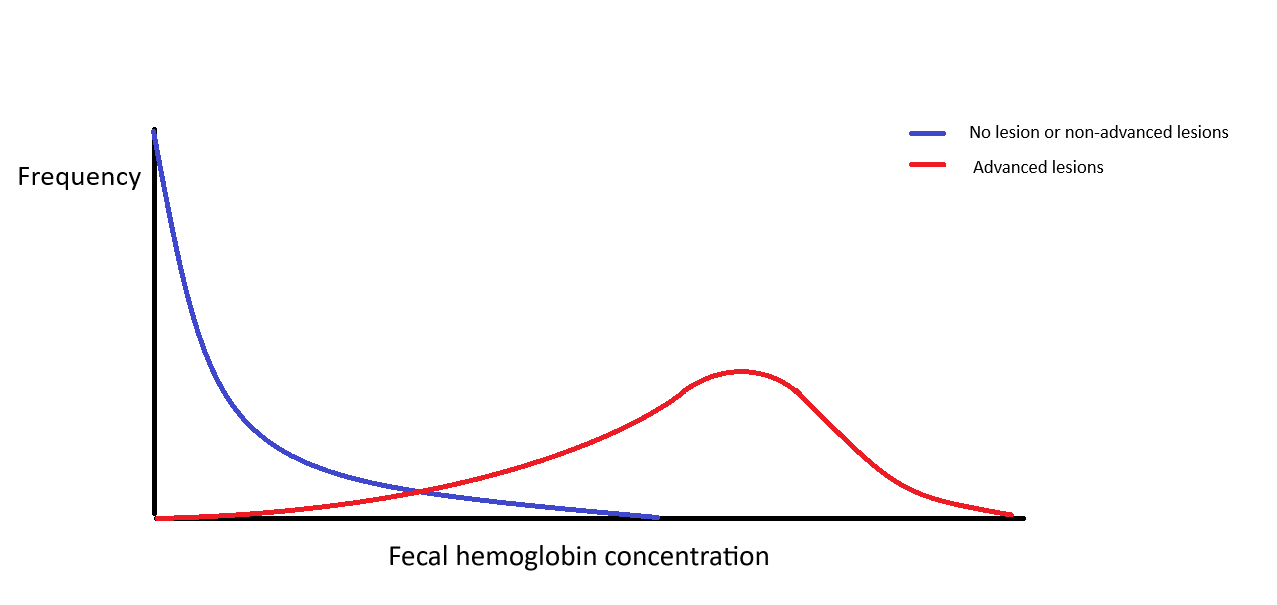


**Supplementary Figure 2** Illustration of the origin of the bimodal distribution of fecal hemoglobin levels.

**Supplement A Model description**

The f-Hb concentration for individual $i$ at screening round $j$, $Y_{ij}$, is modeled as a mixture of a point mass at zero and a negative binomial distribution.

$$Y_{ij}\sim\left\{ \begin{aligned} 0, &with probability \pi_{ij} \\ NegBin(\mu_{ij},\theta), &with probability 1-\pi_{ij} \end{aligned} \right.$$

where

$$\pi_{ij}={logit}^{-1}(\alpha_{0}+\alpha_{1}{Age}_{ij}+\alpha_{2}{Sex}_{i}+\alpha_{3}{NAA}_{ij}+\alpha_{4}{AA}_{ij}+\alpha_{5}{CRC}_{ij} )$$

$\log\left( \mu_{ij} \right)=\beta_{0}+\beta_{1}{Age}_{ij}+\beta_{2}{Sex}_{i}+\beta_{3}{NAA}_{ij}+\beta_{4}{AA}_{ij}+\beta_{5}{CRC}_{ij}+b_{i}$

$b_{i} \sim N(0,\sigma_{b}^{2})$,

with

$\pi_{ij}$ = probability of true zero f-Hb concentration,
$\alpha_{k}$ = regression coefficients for zero-inflation,
$NAA$ = variable used to indicate the presence (1) or absence (0) of a non-advanced adenoma,
$AA$ = variable used to indicate the presence (1) or absence (0) of an advanced adenoma,
$CRC$ = variable used to indicate the presence (1) or absence (0) of colorectal cancer,
$\mu_{ij}$ = mean of the negative binomial distribution for individual $i$ at round $j$,
$\theta$ = dispersion parameter,
$b_{i}$= random intercept capturing between-individual variation and within-individual correlation over time,
$\sigma_{b}^{2}$= variance of the random intercept

The negative binomial probability mass function is defined as:

$$P\left( Y_{ij}=y \right)=\frac{\Gamma\left( y+\theta\right)}{\Gamma\left( \theta\right)y!}\left( \frac{\theta}{\theta+\mu_{ij}} \right)^{\theta}\left( \frac{\mu_{ij}}{\theta+\mu_{ij}} \right)^{y}, y=0,1,2,\ldots$$

with variance: $Var\left( Y_{ij} \right)= \mu_{ij}+\frac{{\mu_{ij}}^{2}}{\theta}$

For values of $Y_{ij}>300$ µg/g, we account for the buffer’s binding capacity limit by substituting the predicted f-Hb value with a draw from a normal distribution $N\left( \mu_{upper},\sigma_{upper}^{2} \right),$ where $\mu_{upper}$​ corresponds approximately to the peak location in the upper tail of the empirical f-Hb distribution from the dataset used for calibration.

The parameters $\alpha_{0,}\alpha_{1}$,$\alpha_{2}$,$\alpha_{3}$*,* $\alpha_{4}, \alpha_{5}, \beta_{0},$ $\beta_{1},$ $\beta_{2}$, $\beta_{3}$, $\beta_{4}, \beta_{5}$, $\theta$, and $\sigma_{b}^{2}$, were calibrated using the Nelder-Mead algorithm.

The parameters $\mu_{upper}$ and $\sigma_{upper}^{2}$ were manually calibrated.

**Supplementary Table 1** Calibration targets for the positivity rate (proportion of positive FITs among all FITs) and detection rates (proportion of FITs in which a lesion is detected among all FITs) for CRC, advanced adenomas, and non-advanced adenomas at different positivity cutoffs. Detection rates were calculated as the positivity rate multiplied by the positive predictive value (PPV). This approach adjusts for the fact that not all individuals with a positive FIT will undergo colonoscopy, while we assumed 100% participation to diagnostic colonoscopy in the model. These targets were derived from data collected in the first half of 2014 as part of the Dutch national CRC screening program, which invited individuals ages 55-75 for biennial screening with a positivity cut-off of 15 µg/g.

| **Positivity cutoff (µg/g)** | **Positivity rate** | **Detection rate CRC** | **Detection rate advanced adenomas** | **Detection rate**  **non-advanced adenomas** |
| --- | --- | --- | --- | --- |
| 15 | 0.11 | 0.009 | 0.047 | 0.027 |
| 20 | 0.10 | 0.009 | 0.043 | 0.023 |
| 30 | 0.08 | 0.008 | 0.038 | 0.017 |
| 40 | 0.07 | 0.008 | 0.034 | 0.014 |
| 50 | 0.07 | 0.008 | 0.032 | 0.012 |
| 60 | 0.06 | 0.008 | 0.029 | 0.010 |

Abbreviations: µg/g: microgram hemoglobin per gram feces, CRC: colorectal cancer

| 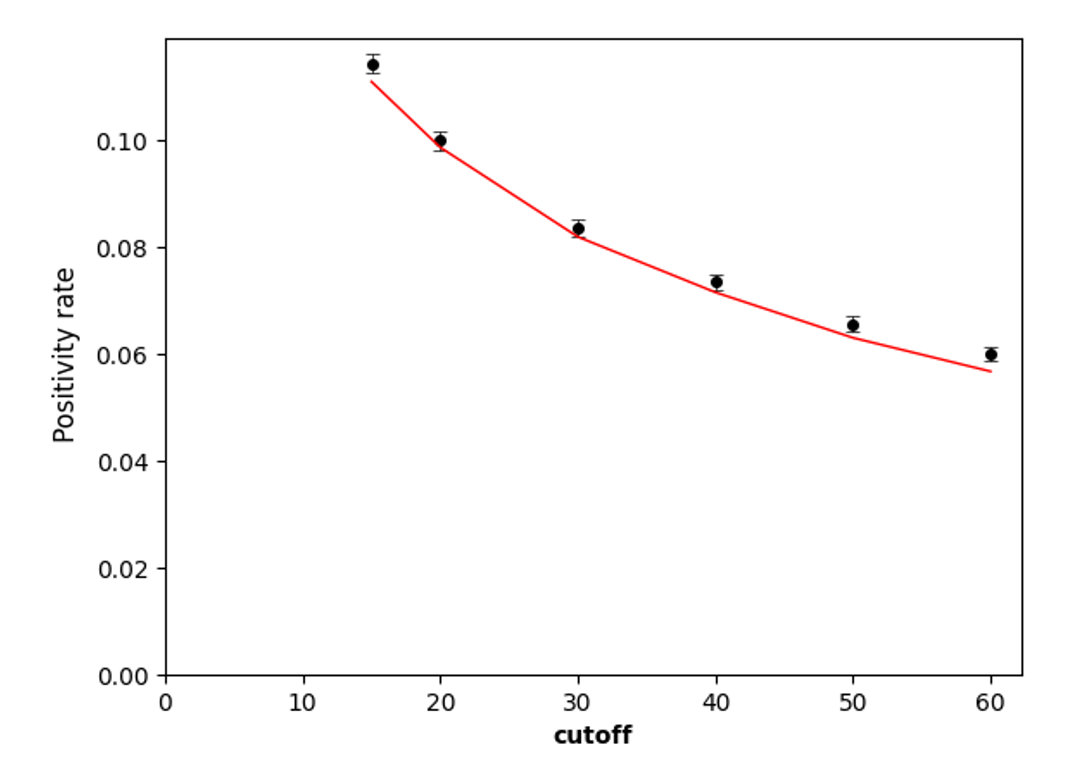  A | 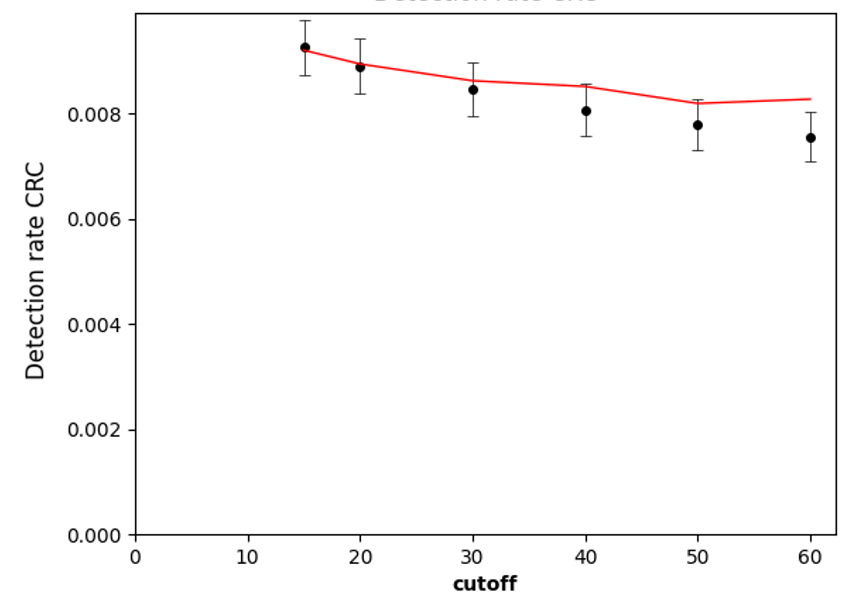  B |
| --- | --- |
| 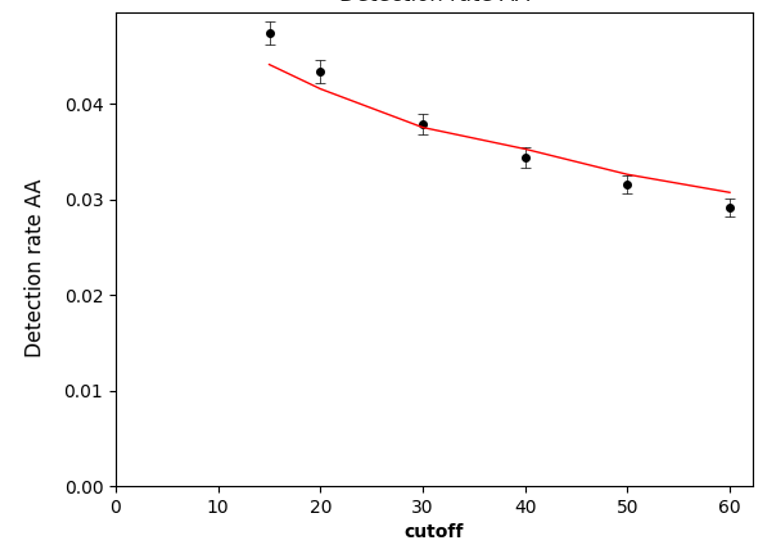  C | 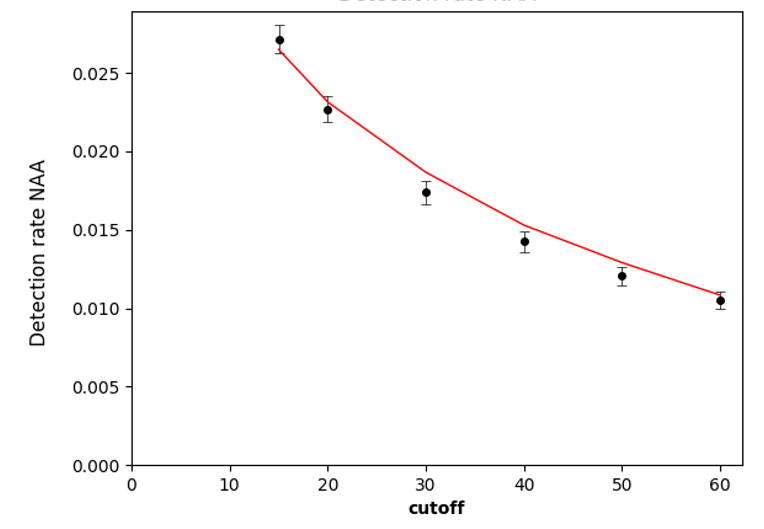  D |
| 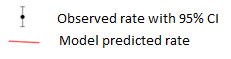 |  |

***Supplementary Figure 3*** *The predicted vs observed positivity rate (A) and detection rates for colorectal cancer (B), advanced adenomas (C) and non-advanced adenomas (D) along with the 95% confidence interval. The observed positivity and detection rates* *were derived from data collected in 2014 as part of the Dutch national CRC screening program, which invited individuals ages 55-75 for biennial screening with a positivity cut-off of 15 µg/g. In the model, 100% participation in screening and diagnostic colonoscopy was assumed. To account for non-adherence to colonoscopy in the observed data, detection rates are calculated as the positivity rate multiplied by the positive predictive value. Abbreviations: CRC: colorectal cancer, AA: advanced adenoma, NAA: non-advanced adenoma*

**Supplementary Table 2** Fecal hemoglobin concentrations at selected percentiles for the simulation and observational data of the Dutch colorectal cancer screening program (mid 2014-2020). The screening program targeted individuals aged 55-75 years for biennial screening and used a FIT positivity cut-off of 47 µg/g. µg/g: microgram hemoglobin per gram feces, CRC: colorectal cancer.

| Percentile | **MISCAN-Colon with quantitative FIT (in µg/g)** | **Observational data (in µg/g)** |
| --- | --- | --- |
| 0.50 | 0 | 0 |
| 0.75 | 0 | 0 |
| 0.80 | 0 | 0 |
| 0.85 | 2 | 0 |
| 0.90 | 9 | 6 |
| 0.95 | 37 | 42 |
| 0.99 | 219 | 215 |

*
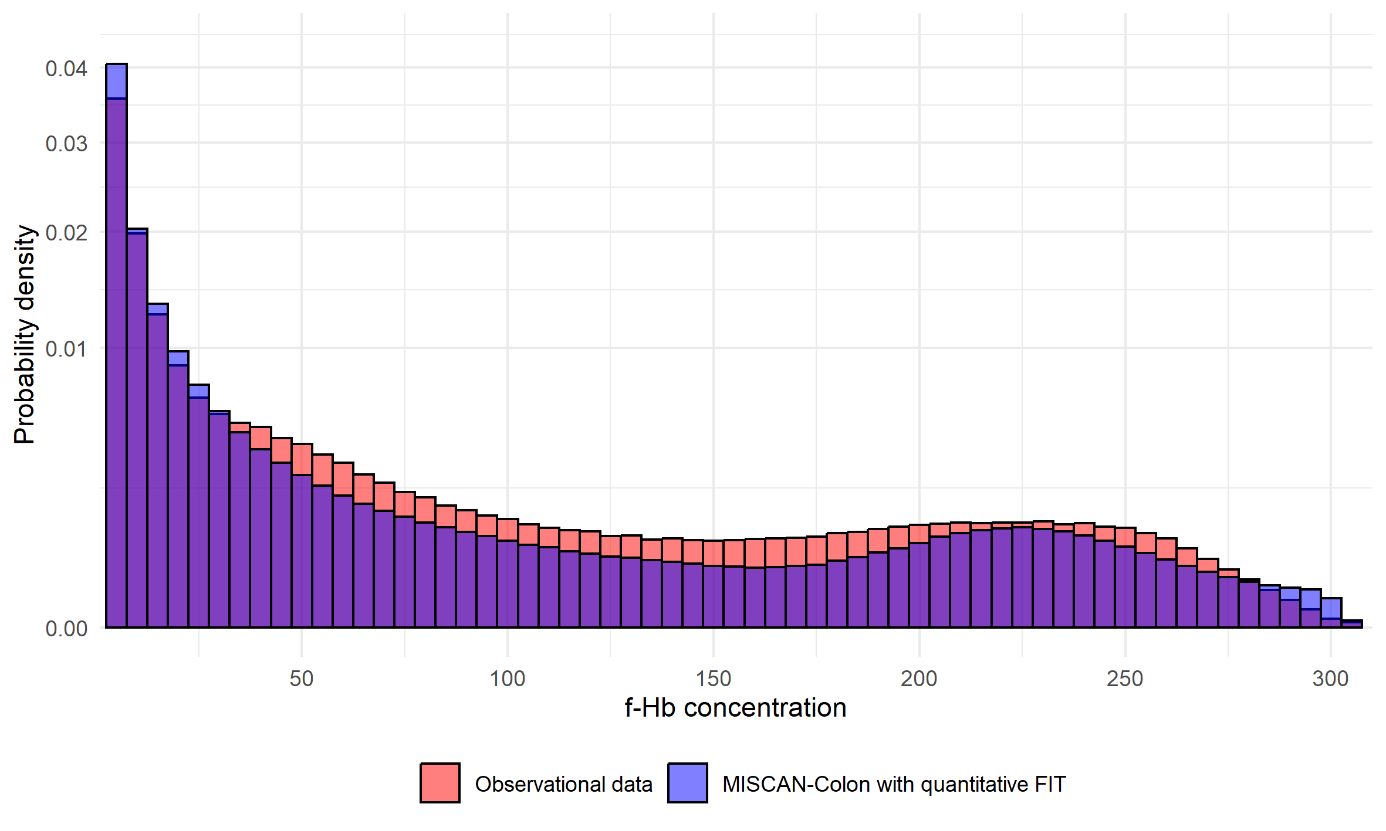
*

***Supplementary Figure 4*** *The distribution of non-zero fecal hemoglobin (f-Hb) concentrations for the simulation and observational data of the Dutch colorectal cancer screening program (mid 2014-2020).  The screening program targeted individuals aged 55-75 years for biennial screening and used a FIT positivity cut-off of 47 µg/g. The purple area represents the overlap between the two distributions. FIT: fecal immunochemical test. F-Hb: fecal hemoglobin.*


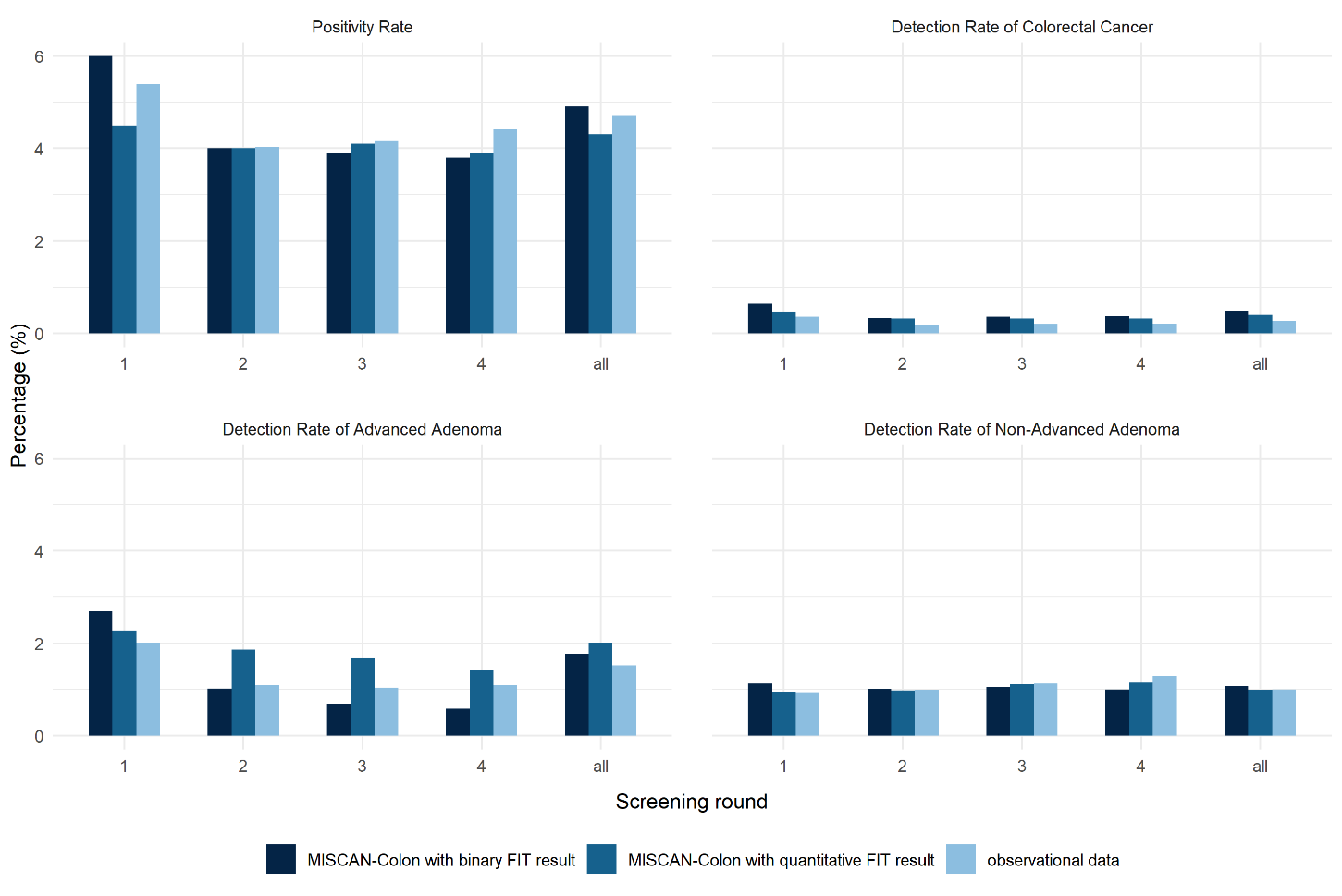


**Supplementary Figure 5** Validation of the positivity and detection rates of the MISCAN-Colon model with f-Hb module (quantitative FIT result) against the MISCAN-Colon outcomes with binary FIT result and observational data of the Dutch colorectal cancer screening program (2014-2020) across various screening rounds. The screening program targeted individuals aged 55-75 years for biennial screening and used a FIT positivity cut-off of 47 µg/g. µg/g: microgram hemoglobin per gram feces; FIT: fecal immunochemical test.

*
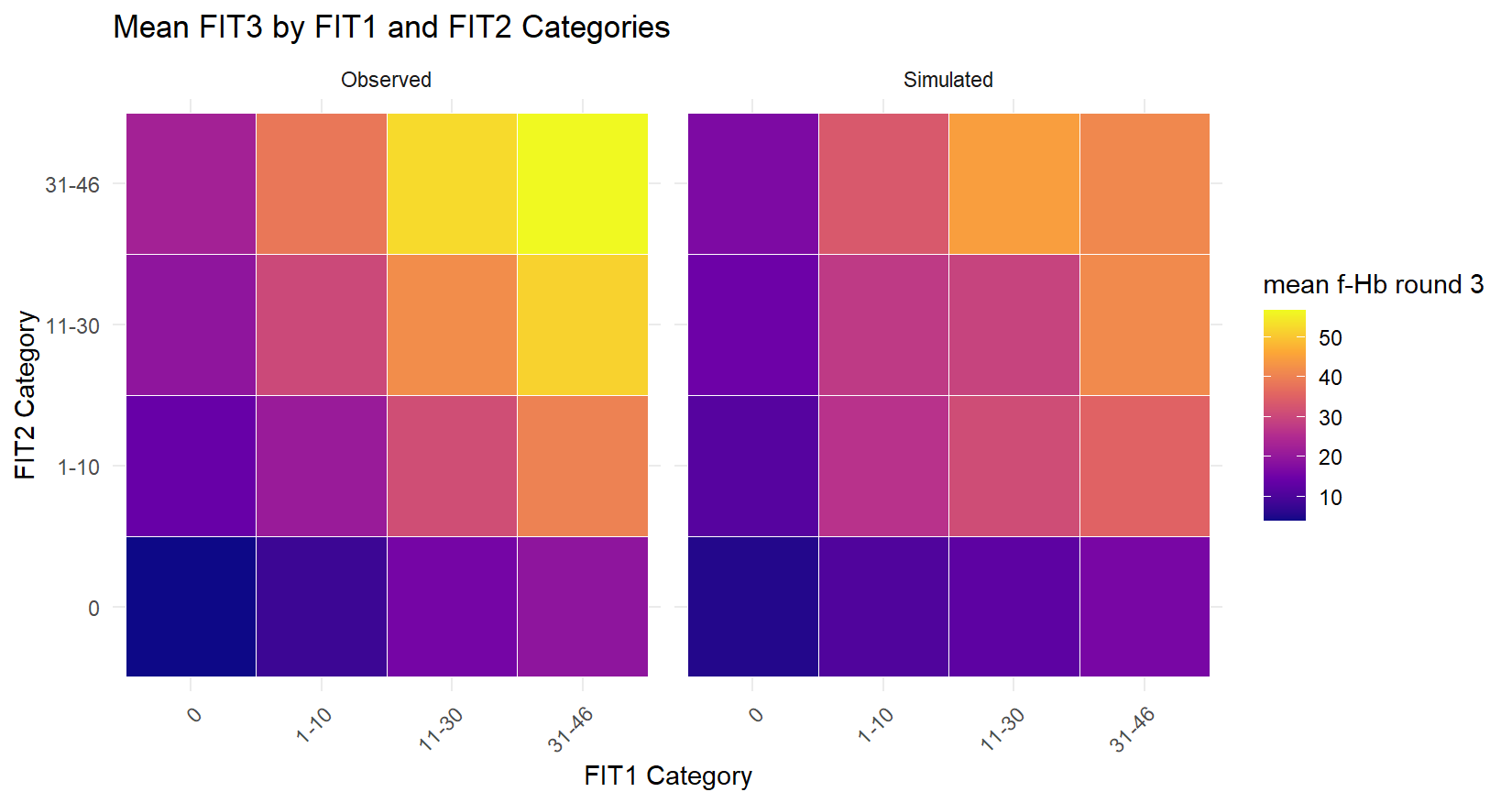
*

**Supplementary figure 6** Mean f-Hb concentration in screening round 3 stratified by f-Hb concentration in screening round 1 and 2. For this analysis only individuals with results in all three screening rounds were selected. The observational data were obtained from the Dutch national colorectal cancer screening program from mid-2014 to 2020. The screening program targeted individuals aged 55-75 years for biennial screening and used a FIT positivity cut-off of 47 µg/g. FIT: fecal immunochemical test.

**
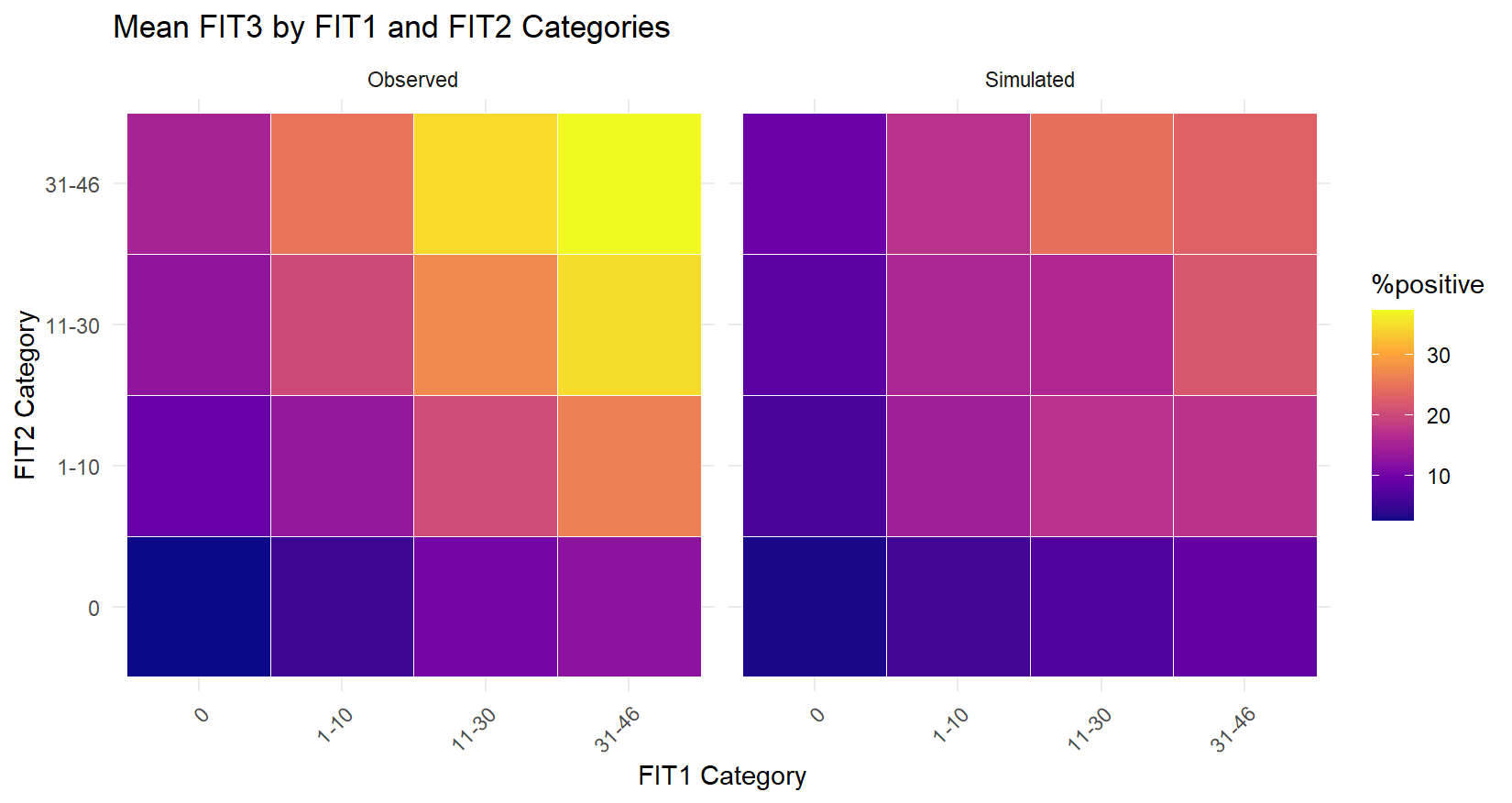
**

**Supplementary figure 7** Positivity rate in screening round 3 stratified by f-Hb concentration in screening round 1 and 2. For this analysis only individuals with results in all three screening rounds were selected. The observational data were obtained from the Dutch national colorectal cancer screening program from mid-2014 to 2020. The screening program targeted individuals aged 55-75 years for biennial screening and used a FIT positivity cut-off of 47 µg/g. FIT: fecal immunochemical test

 
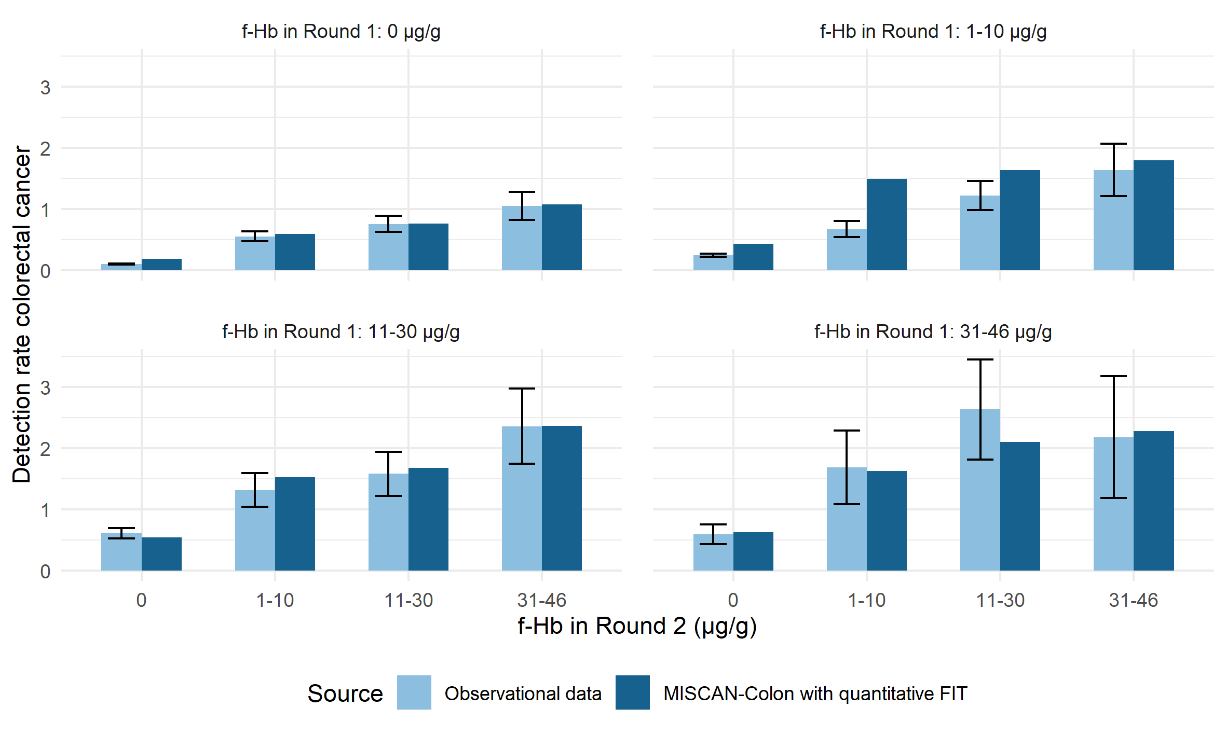


**Supplementary Figure 8** Detection rate for colorectal cancer in screening round 3 stratified by fecal hemoglobin (f-Hb) concentration in screening rounds 1 and 2. The observational data were obtained from the Dutch national colorectal cancer screening program from mid-2014 to 2020. The screening program targeted individuals aged 55-75 years for biennial screening and used a FIT positivity cut-off of 47 µg/g. µg/g: microgram hemoglobin per gram feces; FIT: fecal immunochemical test. F-Hb: fecal hemoglobin


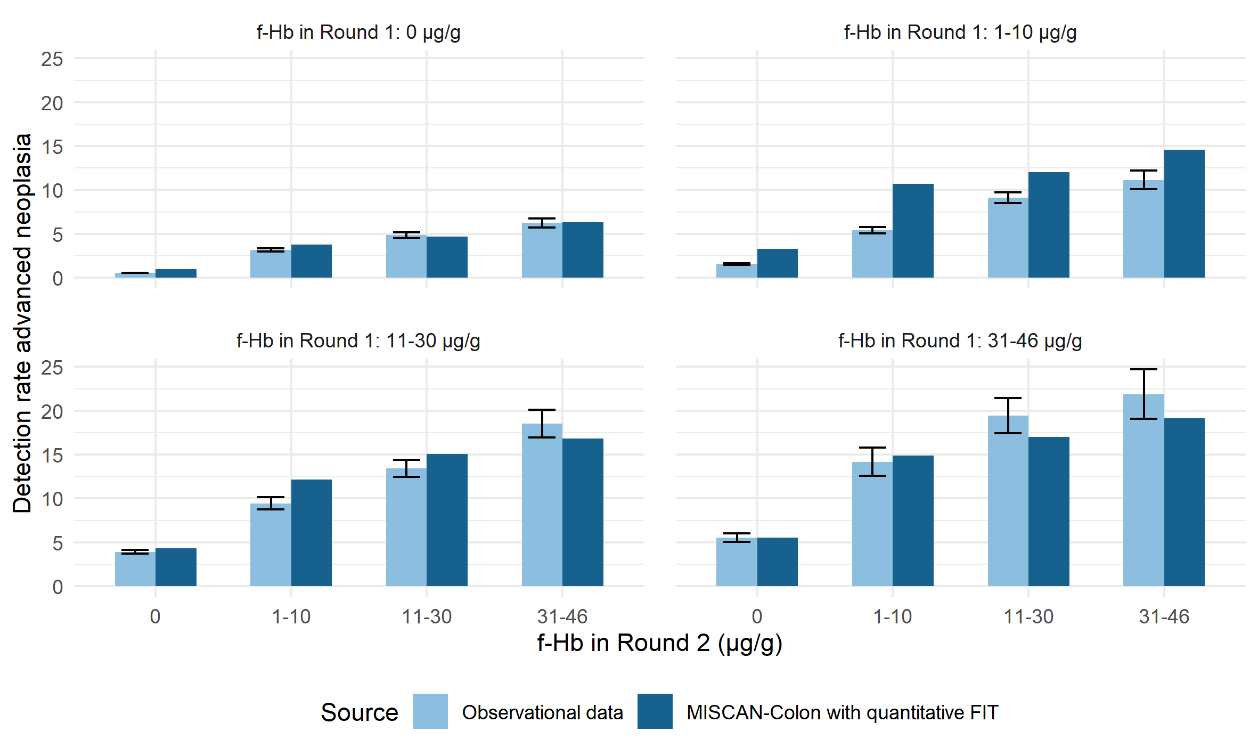


**Supplementary figure 9** Detection rate for advanced neoplasia in screening round 3 stratified by fecal hemoglobin (f-Hb) concentration in screening rounds 1 and 2. The observational data were obtained from the Dutch national colorectal cancer screening program from mid-2014 to 2020. The screening program targeted individuals aged 55-75 years for biennial screening and used a FIT positivity cut-off of 47 µg/g. µg/g: microgram hemoglobin per gram feces; FIT: fecal immunochemical test. F-Hb: fecal hemoglobin
